# Biological Fractionation of Lead Isotopes in Sprague-Dawley Rats Lead Poisoned via the Respiratory Tract

**DOI:** 10.1371/journal.pone.0052462

**Published:** 2012-12-26

**Authors:** Jing Wu, Duojian Liu, Qing Xie, Jingyu Wang

**Affiliations:** 1 School of Public Health, Peking University, Haidian District, Beijing, People’s Republic of China; 2 Center of Medical & Health Analysis, Peking University, Beijing, People’s Republic of China; 3 School of Chemical Engineering and Technology, Hebei University of Technology, Tianjin, People’s Republic of China; University of Kentucky, United States of America

## Abstract

**Objectives:**

It was considered that lead isotope ratios did not change during physical, chemical, or biological processes. Thus, lead isotope ratios have been used as fingerprints to identify possible lead sources. However, recent evidence has shown that the lead isotope ratios among different biological samples in human are not always identical from its lead origins in vitro. An animal experiment was conducted to explore the biological fractionation of lead isotopes in biological systems.

**Methods:**

24 male Sprague-Dawley (SD) rats were divided into groups that received acute lead exposure (0, 0.02, 0.2, or 2 mg/kg body weight of lead acetate) via the respiratory route every day for 5 days. Biological samples (i.e., blood, urine, and feces) were collected for comparison with the lead acetate (test substance) and the low-lead animal feed (diet) administered to the rats. The lead isotope ratios were determined by inductively coupled plasma mass spectrometry (ICP-MS).

**Results:**

There are significant differences (*p*<0.05) in lead isotope ratios between blood, urine, and feces. Moreover, a nonlinear relationship between the blood lead concentration and the blood lead isotope ratios was observed. There is also a threshold effect to the fractionation function. Only the blood isotope ratio of ^204^Pb/^206^Pb matches the test substance well. As for feces, when ^204^Pb/^206^Pb ratio is considered, there is no significant difference between feces-test substance pairs in medium and high dose group.

**Conclusions:**

The biological fractionation of lead isotopes in SD rats was observed. Moreover, there might be a threshold for the biological fractionation of lead isotopes which is depending on whole blood lead level. It is considered to be more reliable that we compared the isotope ratios of potential lead hazards with both blood and feces lead fingerprints especially for ^204^Pb/^206^Pb ratio under high-dose exposure.

## Introduction

Historical long problem of public exposure to lead is still a significant global public health concern due to its cumulative toxicity [1]. In order to reduce human health risks of lead pollution effectively, it is important to pinpoint the sources of lead accurately.

Lead has four naturally occurring isotopes: ^204^Pb, ^206^Pb, ^207^Pb, and ^208^Pb. ^206^Pb, ^207^Pb and ^208^Pb are the radiogenic product of disintegration of ^238^U, ^235^U and ^232^Th nuclides, while ^204^Pb is non-radiogenic [2], [3]. When lead is extracted from its ore, its isotopic composition was frozen at the time [3]. The comparative abundance of the four natural isotopes is characteristic for different lead sources and the isotope ratios can serve as a ‘fingerprint’ of the lead from that source [2], [4], [5]. As Cheng and Hu [5] reported that lead undergoes a minimal mass-dependant isotope fractionation in natural physical, chemical, and biological processes due to its large atomic weight. Thus, these natural variations in lead isotope ratios can be used to determine the origins of lead. To date, lead isotope analysis is the most effective method for the provenance study of lead pollutants. This technique is commonly used to identify human lead exposure sources in various disciplines, including agriculture, archaeology, and environmental science [6]–[8].

However, recent evidence has demonstrated that there were significant differences in lead isotope ratios between blood and urine and between blood and hair in humans [9], [10]. These phenomena suggest that the lead isotopic fingerprints of biological samples in vivo may not always be consistent with the lead origins in vitro. Anthropogenic lead pollution usually originates from mining, smelting, industrial uses, waste incineration, coal burning, and leaded gasoline; due to the complex environment of human lead exposure, it is difficult to identify lead origins by contamination investigation [5]. Thus, analysis of the factors that affect the differences in the lead isotope ratios among the biological samples from the same person is challenging. Moreover, a number of factors could cause a redistribution of lead within the body after lead exposure [11]. It is not known whether there is fractionation of the four stable isotopes of lead during the absorption, distribution, metabolism, and excretion processes in the body. There are few published reports concerning the differences in the lead isotope ratios among biological samples from the same individual, and thus the mechanisms for these differences are still unclear.

Humans absorb lead through the gastrointestinal and respiratory tracts, but rarely through the cutaneous pathway unless there is abrasion or shot wound in the skin [12]–[14]. Although phasing out of leaded gasoline has been carried out in many countries, strong lead sources in air still exist [1], [15]. Moreover, lead absorption through the respiratory route is more rapid and complete than any other routes [16]. Therefore, an animal experiment focusing on the respiratory tract of lead exposure was designed to explore four questions. 1. Is there biological fractionation of stable lead isotopes in lead-poisoned rats? 2. What is the relationship between lead isotope ratios and blood lead level? 3. Is there a threshold effect to the biological fractionation function? 4. Which sample is the most reliable and suitable biomarker to trace environmental lead sources through respiratory route?

## Materials and Methods

### Chemicals and Reagents

Deionized water (conductivity 18 MΩ•cm, GN-RO-100 purification system, Beijing Shuangfeng Science & Technology Development Co., Ltd.), nitric acid (UP, Suzhou Crystal Clear Chemical Co., Ltd.), ammonium hydroxide (UP, Beijing Chemical Reagent Research Institute), perchloric acid (GR, Tianjin Dongfang Chemical Plant), standards 1000 ppm Pb (National Research Center for Certified Reference Materials, Beijing), SRM 981 (National Institute of Standards and Technology, NIST, USA), lead acetate trihydrate (AR, Guangdong Xilong Chemical Engineering Co., Ltd.), anhydrous diethyl ether (AR, Sinopharm Chemical Reagent Co., Ltd.), and chloral hydrate (AR, Sinopharm Chemical Reagent Co., Ltd. ) were obtained from commercial sources. Standard solutions were prepared daily from the stock solutions in polyethylene bottles with 1% (v/v) HNO_3_ solution. To prevent contamination, all quartz vials and other laboratory ware were soaked in 50% (v/v) HNO_3_ for at least 12 h, rinsed 20 times with deionized water, and air-dried.

### Animal Experiments

#### Ethics statement

All experimental procedures with animals were in strict accordance with the recommendations in the Guide for the Care and Use of Laboratory Animals and approved by the Committee on the Ethics of Animal Experiments of the Peking University Health Science Center (Permit Number: LA2012-60).

#### Animals

Twenty-four physically healthy male Sprague-Dawley (SD) rats, weighing 210−250 g, were used for the study; SD rats were obtained from the Department of Laboratory Animal Science, Peking University Health Science Center. All the animals were maintained on the standard laboratory specific pathogen-free (SPF) low-lead diet and given water ad libitum.

#### Exposure to lead

A lead acetate solution, the source of lead, was diluted with deionized water to obtain the different concentrations appropriate for the exposure dose for each group. After adaptive feeding for 3 days, the rats were randomly divided into 4 groups (n = 6 per group), including a control group and three experimental groups. All rats were anesthetized through intraperitoneal injection of 300 mg/kg body weight of 10% (w/v) chloral hydrate solution. Additional doses were administrated as necessary to keep the animals completely anesthetized. Anesthetized animals were then placed under a heating light to maintain normal body temperature.

Intratracheal administration was accomplished by attaching a 0.1 mL syringe to the endotracheal tube. The low dose, medium dose, and high dose experimental groups received 0.02, 0.2, and 2 mg/kg body weight of lead acetate, respectively, while the control group received a single endotracheal injection of equal volume of deionized water; doses were given every day for 5 days.

#### Sampling

Twelve hours after the fifth dose of lead, all rats were placed individually in stainless steel metabolic cages to allow for separate collection of urine and feces without contamination. Rats were supplied with water ad libitum, but were deprived of food. Urine samples were collected continuously for 12 h in 50 mL screw cap polyethylene tubes. The feces samples were collected in polyethylene bags. At sacrifice, rats were anesthetized with ether and ∼4 mL whole blood was collected into a low-lead lithium heparin tube to prevent coagulation. The blood samples were stored at 4°C, and the urine and feces samples were stored at −20°C.

### Sample Preparation

For total digests, portions of the sample (0.5 mL whole blood, 2 mL urine, 0.2 g feces, 0.5 g feed) were weighed in open quartz vials, combined with 5 mL mixed acid (HNO_3_ and HClO_4_ at a 20∶1 volume ratio), and leached in the acidic solution at room temperature for at least 12 h. The quartz vials (each with a quartz ball to keep the acid condensation and reflux) were heated on a hot plate until the solutions became clear. The remaining solids were re-dissolved and transferred to 5 mL centrifuge tubes with 4 mL deionized water. An equal volume of mixed acid was digested simultaneously and used as blank.

### Analysis by Inductively Coupled Plasma Mass Spectrometry (ICP-MS)

To reduce the matrix effect, the separation of Pb from the complex matrix of blood, urine, feces, and food was followed by isotopic analysis by ICP-MS [17]. Measurements were carried out in a clean room with Class 1000-filtered air. Analyses were performed by ICP-MS on a PerkinElmer ELAN DRC (USA). The operating parameters for ICP-MS were described in Wu et al. [18]. The ^204^Pb signal was corrected for ^204^Hg via the ^202^Hg signal using the equation: −0.230074×^202^Hg. The correction was suggested and performed by the ELAN version 2.4 software.

The relative standard deviation (RSD) (n = 6) of 10 ng/mL lead standard solution is shown in Table 1. Standard reference material (SRM) 981 natural lead (isotopic) from NIST was applied for normalization of lead isotope signals. All observed ratios were corrected using the average of the observed isotope ratios for SRM 981. The SRM was prepared daily with the same HNO_3_ solution used to prepare the study samples and was analyzed before and after the samples of every three rats. The value of all SRM analyses was used for calculation of the correction factors. Corrected atom percents of each lead isotope were calculated by applying the correction factors to the observed isotope ratios using Microsoft Excel after data acquisition.

**Table 1:** Precision by ICP-MS on Pb standard solution isotope ratios.

Parameter	RSD (%)
^204^Pb/^206^Pb	0.11
^207^Pb/^206^Pb	0.05
^208^Pb/^206^Pb	0.14

### Statistical Analysis

Statistical analyses were performed using SPSS 16.0 for Microsoft Windows. A one-way ANOVA was used for comparison of isotope ratios between different dose groups and different samples. Pair-wise Comparison was performed using the LSD method and the Tamhane’s T2 method. The independent samples t-test was used for comparison of isotope ratios between biological samples and the ingested lead. When the datasets were not normally distributed, nonparametric tests were used. A *p* value of less than 0.05 was considered statistically significant.

## Results

### Comparison of Lead Isotope Ratios among Blood, Urine and Feces

In this study, the ^204^Pb/^206^Pb ratio of the feces samples was significantly different from that of the blood and urine samples in the control group, in which the low-lead diet was the only source of lead (Table 2). In the experimental groups, the administered lead acetate (test substance) was the major source of lead exposure. Significant differences were found between blood-urine pairs as well as urine-feces pairs in both the medium- and high-dose group. However, the isotope ratio of feces and blood were isotopically indistinguishable.

**Table 2:** Statistical significance of differences in ^204^Pb/^206^Pb for test substance, diet, blood, urine, and feces in different dose groups (n = 6) of respiratory lead exposure.

	Mean ± SD				
Dose group	Blood	Urine	Feces	Test Substance	Diet
Control	0.0562±0.0008^g^	0.0560±0.0003^g^	0.0548±0.0004^a,b^	0.0551±0.0001	0.0546±0.0003
Low	0.0553±0.0005^c,g^	0.0554±0.0004^g^	0.0550±0.0001^c,f,g^		
Medium	0.0554±0.0004^c,g^	0.0560±0.0005^a,f,g^	0.0553±0.0001^b,g^		
High	0.0552±0.0003^c,g^	0.0557±0.0003^a,f,g^	0.0553±0.0003^b,g^		

Note: Difference are significant when *p*<0.05 level.

a, b A significant difference with blood and urine, respectively.

c, d, e A significant difference with control group, low dose group and medium dose group, respectively.

f, g A significant difference with test substance and diet, respectively.

For ^207^Pb/^206^Pb (Table 3), significant differences were found between urine-feces pairs in each group, including the control group and all of the experimental groups. The isotope ratio of blood differed from that of urine only in high-dose group. When we compared the ratio between blood and feces, significant differences were observed in all groups except the high dose group.

**Table 3:** Statistical significance of differences in ^207^Pb/^206^Pb for test substance, diet, blood, urine, and feces in different dose groups (n = 6) of respiratory lead exposure.

	Mean ± SD				
Dose group	Blood	Urine	Feces	Test Substance	Diet
Control	0.8789±0.0058^g^	0.8741±0.0051^g^	0.8556±0.0070^a,b^	0.8592±0.0016	0.8516±0.0032
Low	0.8721±0.0036^c,f,g^	0.8716±0.0050^f,g^	0.8583±0.0027^a,b,g^		
Medium	0.8673±0.0039^c,f,g^	0.8714±0.0045^f,g^	0.8606±0.0012^a,b,g^		
High	0.8622±0.0021^c,d,e,f,g^	0.8700±0.0039^a,f,g^	0.8629±0.0017^b,d,f,g^		

Note: Difference are significant when *p*<0.05 level.

a, b A significant difference with blood and urine, respectively.

c, d, e A significant difference with control group, low dose group and medium dose group, respectively.

f, g A significant difference with test substance and diet, respectively.

For ^208^Pb/^206^Pb (Table 4), significant differences were observed between all the pairs of blood-urine, blood-feces, and urine-feces in each group except the blood-urine pairs in the medium-dose group and the blood-feces pairs in the high-dose group.

**Table 4:** Statistical significance of differences in ^208^Pb/^206^Pb for test substance, diet, blood, urine, and feces in different dose groups (n = 6) of respiratory lead exposure.

	Mean ± SD				
Dose group	Blood	Urine	Feces	Test Substance	Diet
Control	2.1467±0.0081^g^	2.1264±0.0025^a,g^	2.0990±0.0117^a,b^	2.1030±0.0050	2.1015±0.0055
Low	2.1364±0.0035^c,f,g^	2.1272±0.0042^a,f,g^	2.1005±0.0043^a,b,g^		
Medium	2.1238±0.0056^c,d,f,g^	2.1225±0.0068^f,g^	2.1115±0.0049^a,b,d,f,g^		
High	2.1179±0.0056^c,d^	2.1245±0.0045^a,f,g^	2.1162±0.0048^b,d,f,g^		

Note: Difference are significant when *p*<0.05 level.

a, b A significant difference with blood and urine, respectively.

c, d, e A significant difference with control group, low dose group and medium dose group, respectively.

f, g A significant difference with test substance and diet, respectively.

### Comparison of Lead Isotope Ratios between Biological Samples-test Substance and Biological Samples-diet Pairs

**Figure 1:**
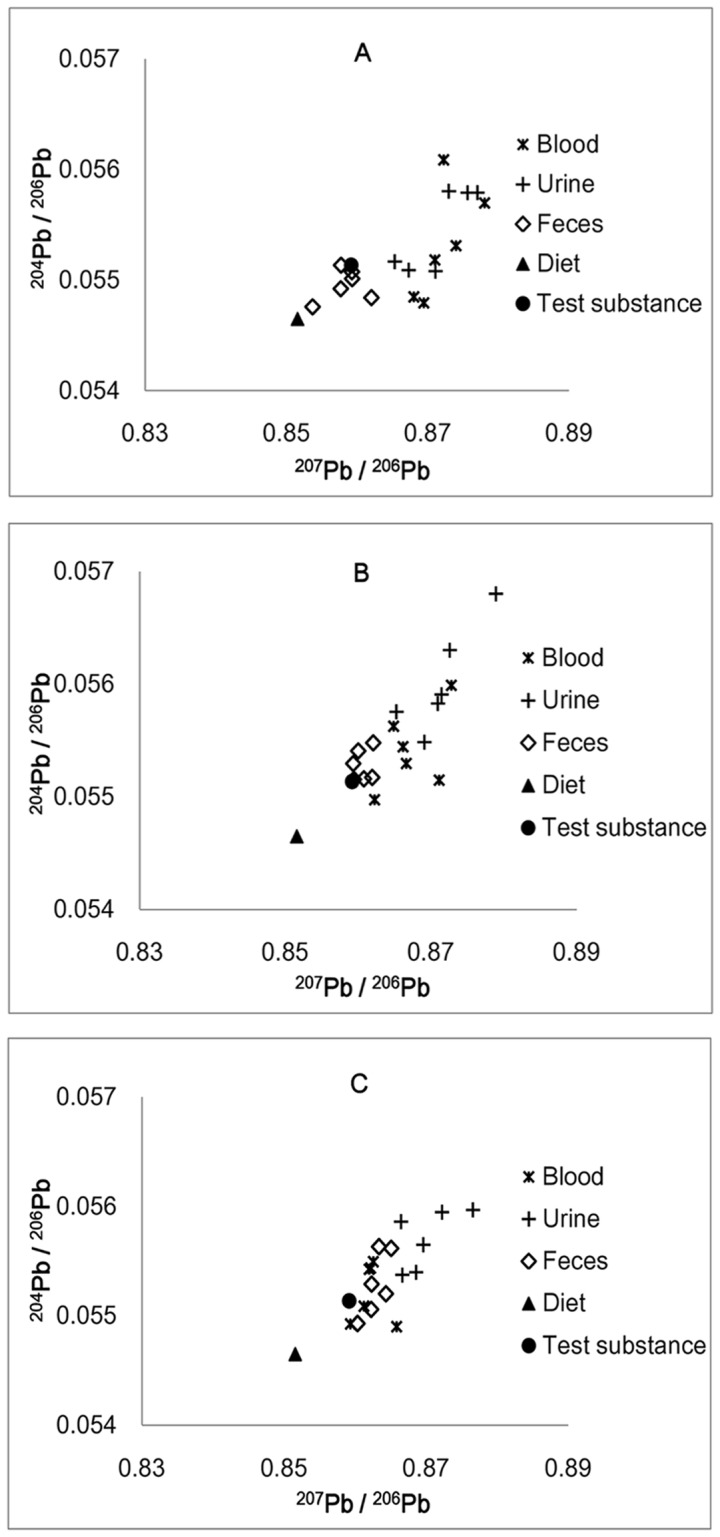
Scatter plot of ^204^Pb/^206^Pb ratio versus ^207^Pb/^206^Pb ratio in different samples. Figure 1A. low-dose group. Figure 1B. medium-dose group. Figure 1C. high-dose group.

Figure 1 shows a two-dimensional distribution of the Pb isotope ratio of ^204^Pb/^206^Pb in various samples against ^207^Pb/^206^Pb in different experimental groups. These results indicate that none of the observed samples matched with the test substance.

In the case of the blood samples, the scattered points became closer to the test substance as the lead dose increased (Fig. 1). For ^204^Pb/^206^Pb, no significant difference was observed between blood-test substance pairs, and the blood isotope ratio in different experimental dose groups did not show significant variations as the dose increased. For the ^204^Pb/^206^Pb isotope ratio alone, the blood samples matched the test substance well (Table 2). By contrast, there were significant differences between the blood-test substance pairs in each dose group when the ^207^Pb/^206^Pb isotope ratio was examined.

The isotope ratios of urine were relatively constant; there was no significant difference between any two dose groups for any isotope ratios. However, there were significant differences between urine and test substance in each dose group for ^207^Pb/^206^Pb (Fig. 1 and Table 3).

For the feces samples, the plots distributed between the test substance and diet in the low-dose group (Fig. 1A) and became closer and closer to the blood samples in the medium- and high-dose group (Fig. 1B–C). The ^204^Pb/^206^Pb isotope ratio showed a significant difference between the feces-test substance pairs in the low-dose group, while no significant difference was observed in the medium- and high-dose groups. When ^207^Pb/^206^Pb was considered, no significant difference was found between the feces-test substance pairs in the low- and medium-dose groups, but there was a significant difference in the high-dose group.

### The Relationship between Blood Lead Concentration and Blood Lead Isotope Ratios

**Figure 2:**
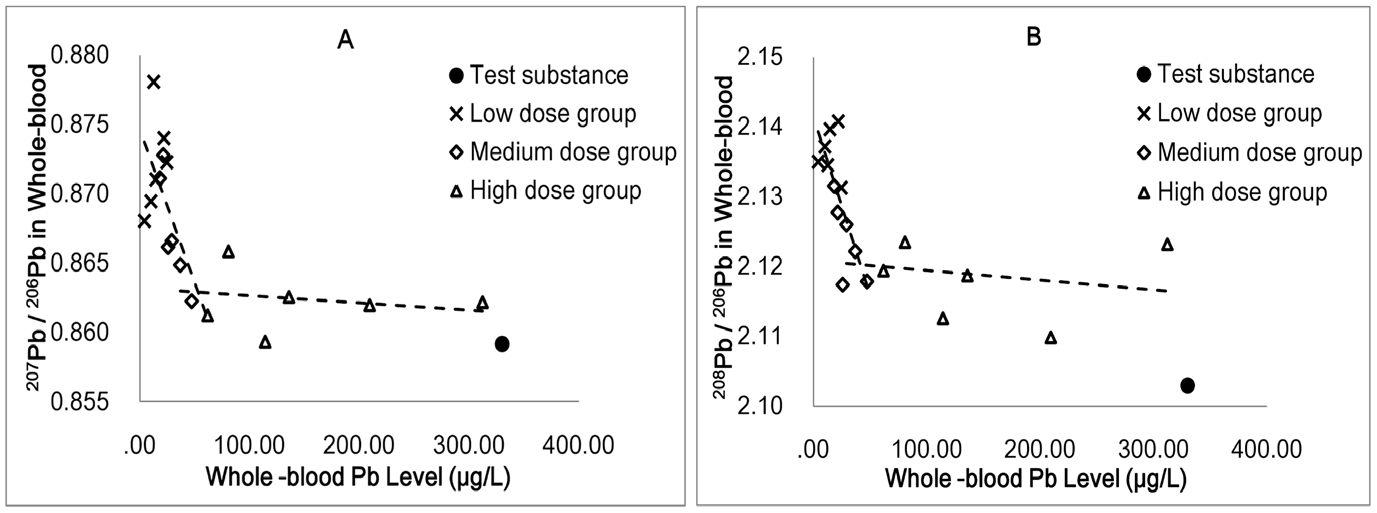
Whole-blood Pb concentration versus isotope ratios in blood for experimental dose groups (n = 18). Figure 2A. isotope ratio of ^207^Pb/^206^Pb. Figure 2B. isotope ratio of ^208^Pb/^206^Pb.

In our study, both the ^207^Pb/^206^Pb and ^208^Pb/^206^Pb ratios in blood were significantly negatively associated with whole-blood lead levels (Fig. 2). An inflexion could be seen for the ^207^Pb/^206^Pb ratio vs. the whole-blood lead level (Fig. 2A). The isotope ratio decreased with the increase in the whole-blood lead concentration and then reached a plateau that tended toward the isotope ratio of the test substance. A similar inflexion can also be observed in Fig. 2B.

## Discussion

Here, we investigated the lead isotope ratios in a variety of biological samples taken from the same individual after lead exposure. We utilized ICP-MS to measure isotope ratios. For this approach to work, different samples possessing distinct isotopic compositions must be distinguishable within the error of the measurement. Gwiazda and Smith [19] concluded that the differences in isotopic composition between two samples should be at least twice the measurement error for each to make reliable conclusions about these differences. According to these conditions and the ICP-MS method used in this study (Table 1), when the differences of the measured isotope ratios for ^204^Pb/^206^Pb, ^207^Pb/^206^Pb, and ^208^Pb/^206^Pb were larger than 0.22%, 0.1%, and 0.28%, respectively, two biological samples could be considered isotopically distinct. The data obtained in this study showed that the ranges of the lead isotopic variation between any two samples were 0.7−2.55%, 0.8−2.7%, and 0.3−2.27% for ^204^Pb/^206^Pb, ^207^Pb/^206^Pb, and ^208^Pb/^206^Pb, respectively. Therefore, the results obtained in this study are reliable.

Because of the high atomic mass of lead and the slight differences in mass of its isotopes, it is assumed by many researchers that no significant degree of fractionation takes place during various metal crafts [5]. As a result, lead isotope ratios vary only according to their geological sources; these natural variations in lead isotope ratios can be used to elucidate the origins of lead. This view was accepted until 1995, when Budd and his colleagues suggested the possibility of lead fractionation [7]. Many experiments have tested the fractionation of lead isotopes during the metallurgical process [7], [20]. In this pilot study, our data indicate that lead isotopic fingerprints had significant differences (*p*<0.05) among different biological samples from the rats lead poisoned via the same tract. These results are consistent with our earlier study, which showed significant differences in lead isotopic compositions between blood and urine and between blood and hair from human [9], [10].

For example, consider the feces samples, in the low-dose group the plots distributed between the test substance and diet (Fig. 1A). According to the following equations [3], [5], we can calculate the fractional contributions of the two sources (i.e., X_D_ and X_TS_), which were the only sources of lead exposure:
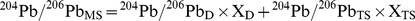
(1)

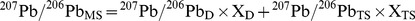
(2)

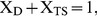
(3)where ‘MS’, ‘D’, and ‘TS’ stand for measured sample, diet, and test substance, respectively, and X is the fractional contribution of the source. In this study, the rats in the low-dose group received ∼4−5 µg lead acetate through intratracheal administration each day and ∼1 µg lead intake through the diet. Theoretically, if there were no other influencing factors, the fractional contribution of the lead in feces and blood should be the same from a given group and between 0 and 100% from each dose group, in accordance with the total amount of lead ingested through the diet and respiratory administration. However, our results did not support this theory. The calculated results of the fractional contribution in feces and blood of the two sources in the different dose groups are listed in Table 5–6. In many cases, the X_TS_ and X_D_ in the blood and feces samples were more than 100% or less than zero.

**Table 5:** The fractional contribution of diet and test substance in different dose group in feces samples.

	^204^Pb/^206^Pb		^207^Pb/^206^Pb	
Dose group	X_D_ (%)	X_TS_ (%)	X_D_ (%)	X_TS_ (%)
Low	36.6	63.4	11.5	88.5
Medium	−29.2	129.2	−18.7	118.7
High	−31.1	131.1	−49.8	149.8

**Table 6:** The fractional contribution of diet and test substance in different dose group in blood samples.

	^204^Pb/^206^Pb		^207^Pb/^206^Pb	
Dose group	X_D_ (%)	X_TS_ (%)	X_D_ (%)	X_TS_ (%)
Low	−37.9	137.9	−171.0	271.0
Medium	−56.9	156.9	−107.1	207.1
High	−15.5	115.5	−39.8	139.8

Therefore, there must be other factors that cause the redistribution of lead isotopes in different biological samples from the lead poisoned SD rats. Generally, there are two possible causes for the differences in isotope abundance [21]. One is radioactive decay, which is not the case in this study, and the other is isotope fractionation, which is caused by small chemical and physical differences between the isotopes of an element. At thermodynamic equilibrium, isotope distributions are strictly governed by relative mass differences among different isotopes of an element; isotopic variations in most biological systems are generally caused by kinetic effects [21]. To date, kinetic fractionation processes have been observed in many bioavailable elements during various biological and chemical processes, including C, O, Ca, Cu, Zn [22]–[27]. Moreover, the thermodynamic equilibrium fractionations should be small and may be overwhelmed by kinetic fractionations at low temperature and in biological systems [21]. Thus, lead may also have a similar fractionation; more studies are required to investigate this hypothesis.

When the rats received a continuous lead exposure, the whole blood lead concentration increased correspondingly. Patel et al. [4] reported that there was no correlation between lead levels and the isotope ratios for the lead from blood of Alaska Natives. Moreover, there are no other published reports that show a relationship between the blood lead concentration and the blood lead isotope ratios. By contrast, in our study, both the ^207^Pb/^206^Pb and ^208^Pb/^206^Pb ratios in blood were significantly negatively associated with whole-blood lead levels. As shown in Figure 2, when the blood lead concentration exceeded the inflexion, the difference of the isotope ratios between the blood and test substance decreased significantly. This phenomenon led us to the following speculation: the inflexion reflected the threshold of the fractionation function of the particular tissues in the rats’ body, at which the blood lead level was ∼50 ng/mL. When the lead level exceeded this threshold, biological fractionation functions of the tissues became abnormal, which resulted in the differences in the isotope ratio between the blood and test substance to be reduced significantly.

As it is observed in our earlier study, there was also a threshold (∼150 ng/mL) for the blood-testis barrier (BTB). If the lead concentration is lower than the threshold, the BTB can protect the testis from lead accumulation; if the blood lead level exceeds the threshold, the lead level in the testis increases significantly and the shape of the sperm become abnormal.

Different thresholds for different biological functions have been already observed in some clinical or subclinical cases, such as proximal tubular cell injury [28], heme synthesis damage [29], decline in children’s IQ [30], intellectual deficits in children [31], and the decrease of arithmetic and reading scores [32]. The values or ranges of the thresholds mentioned above are 600, 150–180, 100, 75, and 50 ng/mL, respectively.

Although the Centers for Disease Control and Prevention defined a blood lead level of 100 ng/mL as the action or intervention level [27], [33], it was also recognized that this blood lead level does not define a threshold for the harmful effects of lead [34]. The safe level of lead in the blood had not yet been identified [35]. However, it should be lower than the values determined in existing studies. Therefore, we introduce the concept of a ‘fractionation functional threshold’. When the blood lead level exceeds this threshold, the fractionation function of the tissues may be damaged and the differences in the isotope ratio between the blood and the test substance would no longer be observed. When the blood lead level is lower than this functional threshold, the fractionation function of the tissues may affect the isotope ratios of biological samples. Therefore, any study that attempts to trace potential lead pollution sources from the environment must consider the blood lead level and the fractionation functional threshold of lead isotopes. Failure to acknowledge the effect of lead biological fractionation could result in a serious under or overestimation of the isotopically different environmental lead sources. However, the results may be relatively accurate when the fractionation function of the tissues is damaged at high blood level; in this case, the isotope ratios of blood become similar to that of the lead source.

Because of the phenomenon of significant differences (*p*<0.05) existing in lead isotope ratios among blood, urine, and feces from the lead poisoned SD rats, due caution should be exercised in choosing the suitable biomarkers to trace the lead sources. In control group, when the diet is the only lead source via gastrointestinal tract (GIT), it is the fingerprints of the feces that match those of the diet well for all the ratios (^204^Pb/^206^Pb, ^207^Pb/^206^Pb, and ^208^Pb/^206^Pb). By contrast, there are significant differences between blood-diet and urine-diet pairs of all the three isotope ratios which indicate that blood and urine are not suitable biomarkers for tracing lead sources when the subjects under a low-lead diet condition only.

In the experimental groups, the ^204^Pb/^206^Pb ratio of blood became undistinguished from the test substance and did not have a significant variation with dose-raising (Table 2). This ratio seems suitable for tracing lead exposure sources from respiratory tract. As for feces, when ^204^Pb/^206^Pb ratio is considered, there is no significant difference between feces-test substance pairs in medium and high dose group. Thus, feces may also be used to identify lead origin when exposure dose exceeded 0.2 mg/kg·d. It is considered to be more reliable that we compared the isotope ratios of potential lead hazards with both blood and feces lead fingerprints especially for ^204^Pb/^206^Pb ratio under high-dose exposure.

However, there are significant differences between urinary Pb isotope ratios and test substances which indicate that the urinary Pb isotope ratios do not match the sources of lead poisoning well. Therefore, urine cannot serve as a reliable biological marker to identify environmental lead pollutant sources ingested via respiratory route.

### Conclusions

There are significant differences (*p*<0.05) in lead isotope ratios between blood, urine, and feces from the lead poisoned SD rats. These results may suggest that there may be a biological fractionation of lead isotopes in the biological system. Moreover, there might be a threshold for the biological fractionation of lead isotopes which is depending on whole blood lead level. When the blood lead level is lower or higher than the threshold, the biological fractionation function may affect the lead isotope ratios in different tissues or may be damaged, respectively. The biological fractionation of lead isotopes should be considered when attempting to trace the potential source of lead pollution in the environment by using stable lead isotope ratios of biological samples. Depending on the present animal experiment, the lead isotope ratios, especially the ^204^Pb/^206^Pb ratio in blood is a significantly sensitive biomarker in tracing lead pollutant which is inhaled through respiratory system. The feces ^204^Pb/^206^Pb ratio could provide reliable source information under a relatively higher exposure dose. The urinary Pb fingerprint is not a suitable biological tracer to identify lead origin in any case.

More research is needed to prove the occurrence of lead isotope fractionation during biological processes and to investigate the effect of the threshold of the biological fractionation function.
